# Design, Synthesis, and Neuroprotective Activity of Phenoxyindole Derivatives on Antiamyloid Beta (Aβ) Aggregation, Antiacetylcholinesterase, and Antioxidant Activities

**DOI:** 10.3390/ph16030355

**Published:** 2023-02-25

**Authors:** Somjate Laivut, Primchanien Moongkarndi, Worawan Kitphati, Pattarawit Rukthong, Korbtham Sathirakul, Kittisak Sripha

**Affiliations:** 1 Department of Pharmaceutical Chemistry, Faculty of Pharmacy, Mahidol University, 447 Sri-Ayudhya Rd., Ratchathewi, Bangkok 10400, Thailand; 2 Department of Microbiology, Faculty of Pharmacy, Mahidol University, 447 Sri-Ayudhya Rd., Ratchathewi, Bangkok 10400, Thailand; 3 Department of Physiology, Faculty of Pharmacy, Mahidol University, 447 Sri-Ayudhya Rd., Ratchathewi, Bangkok 10400, Thailand; 4 Department of Pharmaceutical Technology, Faculty of Pharmacy, Srinakharinwirot University, Nakornnayok 26120, Thailand; 5 Center for Excellence in Plant and Herbal Innovation Research, Strategic Wisdom and Research Institute, Srinakharinwirot University, Nakornnayok 26120, Thailand; 6 Department of Pharmacy, Faculty of Pharmacy, Mahidol University, 447 Sri-Ayudhya Rd., Ratchathewi, Bangkok 10400, Thailand; 7 Unit of Compounds Library for Drug Discovery Mahidol University, 447 Sri-Ayudhya Rd., Ratchathewi, Bangkok 10400, Thailand

**Keywords:** antiamyloid beta aggregation, antioxidant, neuroprotective activity, indole-based scaffold, Alzheimer’s disease

## Abstract

In this investigation, a number of phenoxyindole derivatives were designed, synthesized, and tested for their neuroprotective ability on SK-N-SH cells against Aβ_42_-induced cell death and biologically specific activities involved in anti-Aβ aggregation, anti-AChE, and antioxidant effects. The proposed compounds, except compounds **9** and **10**, could protect SK-N-SH cells at the IC_50_ of anti-Aβ aggregation with cell viability values ranging from 63.05% ± 2.70% to 87.90% ± 3.26%. Compounds **3**, **5**, and **8** demonstrated striking relationships between the %viability of SK-N-SH cells and IC_50_ values of anti-Aβ aggregation and antioxidants. No significant potency of all synthesized compounds against AChE was found. Among them, compound **5** showed the strongest anti-Aβ and antioxidant properties with IC_50_ values of 3.18 ± 0.87 and 28.18 ± 1.40 μM, respectively. The docking data on the monomeric Aβ peptide of compound **5** demonstrated good binding at regions involved in the aggregation process, and the structural feature made it possible to be a superior radical scavenger. The most effective neuroprotectant belonged to compound **8**, with a cell viability value of 87.90% ± 3.26%. Its unique mechanisms for enhancing the protective impact may serve additional purposes since it demonstrated mild biological-specific effects. In silico prediction of CNS penetration shows strong passive penetration ability across the blood–brain barrier from blood vessels to the CNS for compound **8**. In light of our findings, compounds **5** and **8** appeared as potentially intriguing lead compounds for new therapeutic approaches to Alzheimer’s disease. More in vivo testing will be revealed in due course.

## 1. Introduction

AD is characterized by progressive memory loss and a decrease in cognitive ability. Although initial origins leading to AD are unknown, it is clear that the pathogenesis of AD is characterized by two features: extracellular senile plaques and intracellular neurofibrillary tangles (NFTs). Both of them work synergistically to cause neuronal degeneration. When intracellular NFTs result from hyperphosphorylation of tau proteins, which are utilized to support microtubules during the process of mitosis, the transmembrane peptide amyloid precursor protein (APP), which is a precursor to the amyloid beta peptide (Aβ), is cleaved [1,2,3]. APP cleavage starts with either α- or β-secretase, the two competing proteases, and then γ-secretases. The cleavage that begins with β-secretase is the primary pathway in AD and leads to the production of Aβ_40_ and Aβ_42_, which are highly hydrophobic peptides, resulting in the primary components of amyloid plaque formation. Although the protofilaments of Aβ_40_ and Aβ_42_ fibrils have identical numbers of Aβ molecules per cross-β repeat, the longer peptide, Aβ_42_, is more neurotoxic and more prone to aggregation [4]. Starting with oligomers, fibers, and then amyloid plaques, Aβ’s extracellular neurons can exert damaging neuronal and synaptic function, leading to the loss of neuronal cells [5,6,7]. However, the cleavage of APP by α-secretase occurs in the middle of Aβ peptide at residue 17 and leads to the production of nonamyloidogenic fragments of APP and, therefore, inhibiting Aβ production [8]. Gradual loss of neurons caused by amyloid plaques will result in a shortage and imbalance of different neurotransmitters, including decreased acetylcholine synthesis and release, overactivation of N-methyl-D-aspartate receptors, and increased intracellular calcium level and be responsible for the cognitive deficits and behavioral dysfunctions typical of AD [9]. However, the exact mechanism by which Aβ induced neuronal cell death occurs is still unclear. Aβ is often produced in a small number of various lengths of 38–43 amino acids, although typically amino acid lengths are 40 (85–95%) and 42 (5–15%) [10]. The amino acid sequence of different length Aβ is divided into two sections that play a crucial role for turning into β-sheets in amyloid fibrils. The first section represents hydrophobic regions at sequence 16–21 and 29–42, and the second represents hydrophilic regions at sequences 22–28. In the region of 29–42, there are two core regions containing amino acid sequence GxxxG: G = glycine and × = any amino acid which is also involved in proteolysis to form the various lengths of Aβ. All mentioned areas on Aβ involve self-interaction to produce β-sheets stabilized through H-bond, salt bridge (between Asp23 and Leu28), and hydrophobic interaction. In case of blockage of these areas by a small drug-like molecule, Aβ is then unable to self-assemble spontaneously to the hazardous oligomeric structures, which eventually form the mature Aβ fibril plaques [11,12,13,14,15]. Several groups have developed compounds to inhibit various key steps in the amyloidogenic pathway. The beneficial innovations may be preventing the creation of hazardous Aβ peptides that could be self-assembling to be plaques by inhibiting β- or γ-secretase enzymes. The β-secretase (BACE-1) cleaves APP to produce the N-terminus of A peptides. Inhibition of BACE-1 appears to be a viable therapeutic target. Numerous encouraging findings supported this idea, provided new design possibilities, and led to the identification of an intelligent medicine for the treatment of Alzheimer’s disease [16,17,18,19]. Numerous inhibitors of γ-secretase have been discovered, but therapeutic trials for AD were unsuccessful due to insufficient substrate specificity. However, the discovery of substances that can allosterically modulate the protease activity has made γ-secretase an interesting target [20]. As previously indicated, one further strategy has focused on preventing Aβ aggregation with small molecules. With this technique, several lead candidates against Aβ aggregation have been found by screening vast libraries of synthetic and natural chemicals. Natural compound-based molecules of curcumin [21,22,23], catechol [24], polyphenol, epigallocatechin-3-gallate [25], and cinnamon [26] were examined and found to be effective in reducing the Aβ plaques in the brain through their antioxidant and antiaggregation properties [27]. Structural alteration of natural substances, such as curcumin [28] and resveratrol [29], gave highly effective modulation [30] of Aβ aggregation by controlling the harmful oligomeric formation through inhibiting hydrophobic interactions [31]. Newly developed compounds demonstrated the suppression of Aβ aggregation by attaching to the subregion on the monomeric or oligomeric state of the aggregation process [32,33]. We concentrated on indole-based molecules to create novel lead compounds that had an impact on the powerful aggregation process of Aβ [34]. Many compounds that contained the indole moiety could attach to numerous binding sites with high affinity while also displaying their diverse pharmacological properties, such as antibacterial, anti-inflammatory, antioxidant, etc. [34]. Additionally, it was discovered that the indole-based molecule of sulindac sulfide and melatonin provided adequate neuroprotective functions in AD by reducing the toxic oligomers by speeding up the rate of fibril formation [35] and activating α-secretase and suppressing both β- and γ-secretase at the transcription level [36]. Additionally, a number of investigations showed that abnormal acetylcholinesterase activity (AChE) increased Aβ monomer to form fibrils via forming complexes with soluble Aβ and promoting their elongation. In other words, the lack of acetylcholine (ACh) could stimulate Aβ aggregation in like manner. As a result, inhibition of AChE may also prevent Aβ aggregation [37]. The Aβ peptide is a metalloprotein with high-affinity binding of Cu^2+^, Zn^2+^, and Fe^3+^ ions resulting in the production of reactive oxygen species, peroxide, and hydroxyl radical that may lead to oxidative damage on both the Aβ peptide itself and on surrounding area of formation [38]. Many naturally occurring [39,40] and intelligently designed small compounds [40] were then explored as antioxidants.

In this study, we aim to design and create indole-based compounds for multiple anti-Aβ aggregations, anti-AChE, and antioxidant effects to prevent and treat AD.

## 2. Results and Discussion

### 2.1. Synthesis

**Scheme 1:**
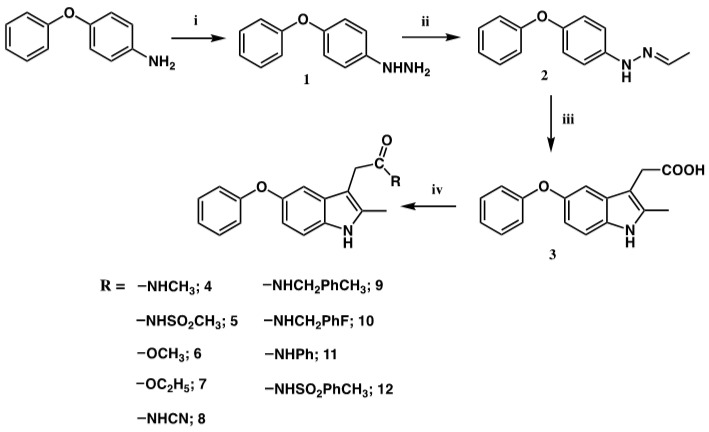
Reagents and conditions: (i) NaNO_2_, HCl, 0 °C, 2 h, H_2_NSO_3_H, SnCl_2_·2H_2_O, HCl, 0 °C, 2 h; (ii) 5 M NaOH, CH_3_CHO, dry DCM, r.t., 2 h; (iii) levulinic acid, glacial acetic acid dry, 80 °C, 3 h; (iv) ROH or RNH_2_ derivatives, EDCI, HOBt, DCM, r.t., 3 h.

All necessary compounds were obtained via four steps as shown in Scheme 1. 4-Phenoxyphenylhydrazine (**1**) was prepared by the diazotization of 4-phenoxyphenylaniline followed by reduction with tin (II) chloride. The formation of imine **2** was achieved using Schiff’s base reaction. Next, hydrazine **1** reacted with acetaldehyde in a basic condition to obtain imine **2**. Fischer indole [41,42,43] synthesis was applied to approach acid **3** by reacting imine **2** with levulinic acid in the presence of glacial acetic acid. Finally, each end product’s ester or amide derivative was achieved through esterification or amidation under mild conditions using EDCI and HOBt as reagents. Scheme 1 depicted the synthesis pathway of desired chemicals.

### 2.2. Biological Activities

#### 2.2.1. Anti-Aβ Aggregation Activity (Thioflavin T Fluorescence Assay: ThT Assay)

Aβ was the primary element of senile plaques in AD derived from APP. It was widely accepted that the pathogenesis of AD began with the gradual buildup of Aβ. This incident set off a series of events that resulted in neurotoxicity, oxidative damage, and inflammatory response. As a result, the antiaggregation activity of our synthesized compounds was assessed using ThT fluorometric assay [44,45] and using curcumin as a positive control. In this experiment, the IC_50_ of each compound was evaluated, and their dose-dependent behavior is listed in Table 1. Curcumin had an IC_50_ of 11.09 ± 1.78 µM, which was strikingly similar to those noted in the literature [46,47].

As shown in Table 1, compounds **4** and **5** displayed the most potent agents in this series with IC_50_ values of 3.26 and 3.18 μM, respectively, against Aβ aggregation and are more effective than curcumin, which was used as a positive control. By focusing on the functional groups in the produced compounds, methyl ester **7** showed better inhibitory action than acid **3** and ethyl ester **6**. Compared to ester and amide derivatives, methyl amide **4** was 4.3-fold more potent than methyl ester **7**. The presence of phenyl and benzyl amide substituents (compounds **9**, **10**, and **11**) decreased their potency. The electron-donating group appeared to reduce the activity more than the electron-withdrawing group compared to compounds **4** and **8**. Similarly, a 1.4-fold reduction of anti-Aβ aggregation was found when *p*-CH_3_ on aromatic benzyl amide derivative (compound **9**) was replaced with *p*-F (compound **10**). Changing the amide to sulfonamide derivatives, as observed in compounds **5** and **12**, considerably enhanced their capacity to prevent the aggregation of Aβ (protein). Except for the sulfonamide derivative of compound **12**, the large substituent amide derivatives (compounds **9**–**11**) decreased the antiaggregation of Aβ protein. This might be proven to have a positive effect on the sulfonamide group’s activity in this series.

#### 2.2.2. Protection of Neuronal SK-N-SH Neuroblastoma against Aβ_42_-Induced Cell Death

The effects of Aβ_42_ on the viability of SK-N-SH neuroblastoma cells were investigated using an MTT reduction test [48]. MTT is a tetrazolium salt that can be reduced to formazan by mitochondria dehydrogenase; the reaction is active only in living cells.

**Figure 1:**
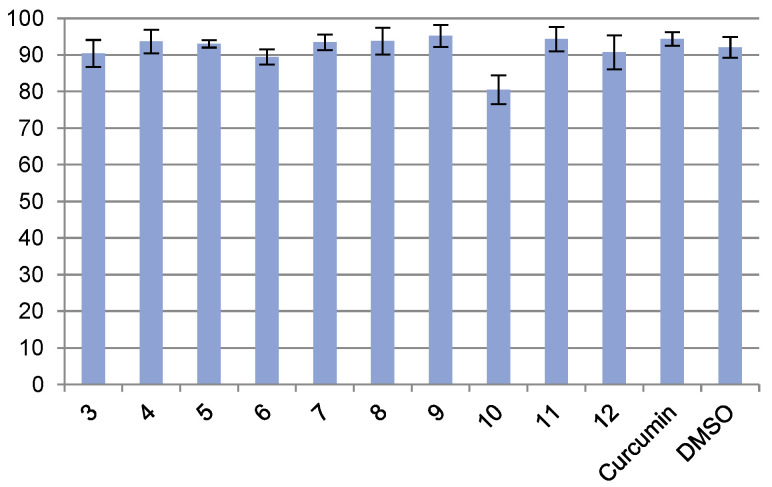
Cytotoxicity of each produced substance, curcumin, and DMSO on SK-N-SH neuroblastoma cells at a concentration of 31.25, 31.25, 62.5, 31.25, 31.25, 62.5, 31.25, 31.25, 15.63, 15.63, and 15.63 µM and 1.25% *v/v*, respectively. The percentages of relative cell viability (mean ± RSD) were calculated at 24 h using MTT assays (*n* = 6) (Y = % viability, X = synthesized compound).

**Figure 2:**
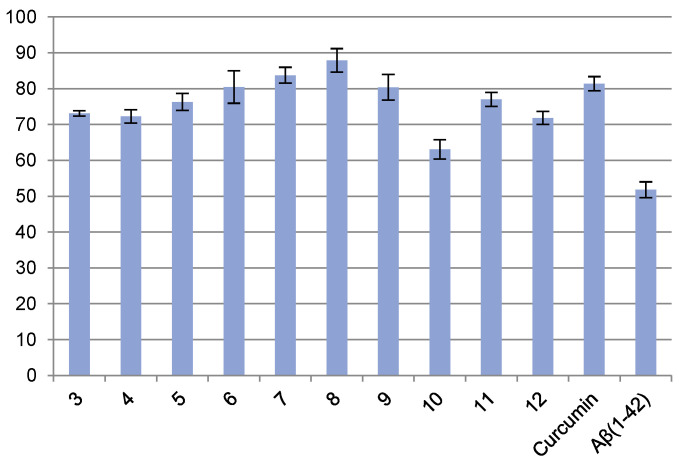
Neuroprotective impact on SK-N-SH neuroblastoma cells of the produced compounds at IC_50_ values of antiaggregation, except compounds **9** and **10**, which were employed at the concentration of 31.25 µM. The percentages of relative cell viability (mean ± RSD) were calculated at 24 h with 25 μM of Aβ_1–42_ using MTT assays (*n* = 6) (Y = %viability, X = synthesized compounds).

DMSO was utilized as a solvent, and our synthesized phenoxyindole derivatives may be harmful to cells at high concentrations. Due to this, the determination of safe concentrations of DMSO at a concentration of 0.625–10% *v/v*, the synthesized compounds, and the positive control, curcumin at a concentration of 0–1000 µM, was performed. The results are illustrated in Table 2 and Figure 1. It was discovered that the percent viability of all synthesized compounds did not show a significant difference (*p* > 0.05), except for compound **10**. The nontoxic concentration of every examined substance was higher than the half-maximal inhibitory concentration for anti-Aβ_42_ aggregations, except compounds **9** and **10**. It indicated that all tested compounds, except **9** and **10**, may be investigated for the neuroprotective effect on SK-N-SH cells at their respective half-maximal inhibitory dose. Compounds **9** and **10** were examined at the nontoxic concentration (31.25 µM). For the suitable concentration of DMSO, we found that it may be utilized in SK-N-SH cells without cellular damage at ≤1.25%. SK-N-SH cells were pretreated for 3 h with the respective half-maximal inhibitory doses of the tested compounds (except **9** and **10**) before incubating with 25 μM Aβ_42_ for 24 h to determine the neuroprotective effect against Aβ_42_-induced toxicity. The relative cell viability (compared with the control) was then assessed. As demonstrated in Table 3 and Figure 2, Aβ_42_ caused a significant reduction in cell viability to roughly 50% of the control. However, the preincubation with the tested compounds protected the cell from Aβ_42_-induced toxicity. Cell viability rose by 50% compared to the group that had only received treatment with Aβ42 due to the considerable protection seen at the IC_50_ for the tested compounds. Compounds **6**–**9** had a greater neuroprotective effect than compounds **1**–**5** and **10**–**12** with a significant difference (*p* < 0.05). However, no discernable difference (*p* > 0.05) between compounds **6**–**9** and curcumin were observed. However, when compared to the control, compound **10** demonstrated a significantly different (*p* < 0.05) protective effect. However, the neuroprotective impact was least in this test since it needed a larger concentration than the IC_50_ value for test evaluation, as previously mentioned. As a result, every molecule created was able to protect the cells against Aβ_42_-induced cell death.

**Figure 3:**
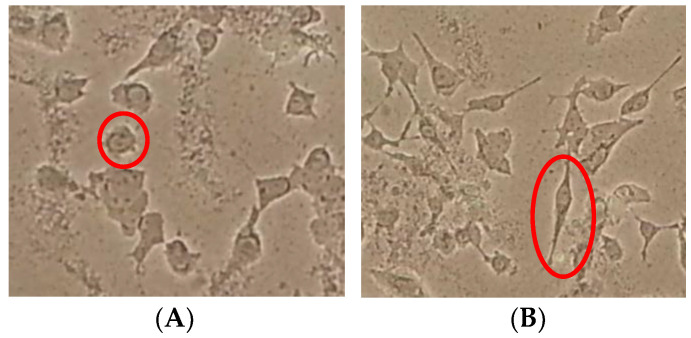
Morphology of SK-N-SH cells treated with Aβ_42_. (**A**) The cells treated with 25 µM Aβ_42_ for 24 h showed a roundup of cell bodies and loss of neuritis. (**B**) The cells treated with Aβ_42_ and compound **5** resulted in healthy cell bodies and neuritis.

The effect of Aβ_42_ in inducing cell death was confirmed using a phase-contrast inverted microscope. After 24 h incubation with 25 µM Aβ_42_, the cells became rounded up and detached from the plate surface, as depicted in Figure 3A, whereas the treated SK-N-SH cells with compound **5** display healthy cell bodies with neurites (Figure 3B).

#### 2.2.3. Antioxidant Activity (DPPH Assay)

Numerous studies have demonstrated that Aβ promotes the production of free radicals. Increased generation of free radicals also results in cytotoxicity from Aβ_42_. This effect might be associated with the Aβ_1–42_ mediated neurotoxicity. Therefore, it was intriguing to research the roles of the synthesized compounds in assisting oxidative stress, thus preventing cell death. By employing vitamin C as a positive control, the antioxidative properties of synthesized compounds were assessed using the DPPH assay [49]. The activities were presented as the IC_50_ in Table 4.

Many compounds showed strong antioxidant activity, such as compounds **3**, **5**, **8**, and **10**, with the IC_50_ value in the range of 28.28–85.97 μM. Among them, compound **5** stood out in terms of antioxidant activity. According to the DPPH assay’s concept, the structure that can produce stable radicals after the departure of H-free radicals will exhibit antioxidant activity. Compounds **6** and **7** contained ethyl and methyl esters in molecules and are unable to produce H-free radicals, so they showed low activities with the IC_50_ values of 190.43 and 144.93 µM, respectively. For the most potent compound **5**, it could be hypothesized that -NH- between the carbonyl and sulfone group could produce a stable radical.

Additionally, this radical was presumably more stable compared to the radical created by compound **12**. Due to a good persistent property of the methane sulfone moiety of compound **5**, the free radical produced moved difficultly in the structure and allowed the radical to remain stable enough to function as an antioxidant. Compound **8** was 3.03-fold less potent than compound **5** due to less stable radicals formed from -NH- between the carbonyl and cyanide group of compound **8**. Both methyl and phenyl amide derivatives, compounds **4** and **11**, demonstrated modest efficacy of antioxidant action with IC_50_ values of 175.74 and 111.07 μM, respectively. Surprisingly, when *p*-CH_3_ (compound **9**) was converted to *p*-F (Compound **10**), the potency was increased 3.08-fold. It might be assumed that a high electronegativity of F could induce the radical generation at -CH_2_- next to the aromatic ring.

#### 2.2.4. Antiacetylcholinesterase Activity (Ellman Assay) [50]

Acetylcholinesterase enzyme (AChE) is a family of serine hydrolases. It can be hindered at the active site or allosteric site. The active site of AChE composes of three parts: an esteric subsite, an anionic subsite, and a hydrophobic region. The allosteric site, called peripheral anionic, is located more than 20 Å away from the active site. Numerous studies indicated that AChE enhanced the Aβ monomer to form fibrils via forming complexes with soluble Aβ through its peripheral anionic site and aided them to elongate. In other words, a deficit of acetylcholine (ACh) could similarly promote Aβ and tau protein aggregation. As a result, inhibition of AChE may also prevent Aβ aggregation. The anti-AChE activity of tested compounds at 100 µM was measured by Ellman assay and using galantamine as a positive control. The outcome is displayed in Table 5.

All developed compounds showed mild to moderate activity for antiacetylcholinesterase activity (Table 5) compared to galantamine, the reference standard. No correlation was found between the antiacetylcholinesterase and antiaggregation activity of the proposed compounds. AChE is not a selective enzyme; in addition to hydrolyzing ACh, it also demethylates and acyclizes other substances similar to AChE, including acrylic esters, anilides, thioesters, amide, and selenoesters [51]. The yellow color of 5-thio-2-nitrobenzoic acid (TNB) is developed from the hydrolysis of the disulfide bond of Ellman’s reagent (5,5-dithio-bis-(2-nitrobenzoic acid) by thiocholine, which is created from the reaction between acetylthiocholine (ACTI) and AChE. As a result, the powerful AChE inhibitor should have a low intensity of yellow color due to the limited generation of TNB. In the case of compound **5**, as shown in Table 5, a negative inhibition value of −5.66% was found. This false negative result probably came from a chemical side reaction that could produce additional TNB in the testing mixture. AChE used the hydroxyl group of Ser-200 as a nucleophile to attack the carbonyl group of ACTI and produced thiocholine. It was most likely brought on by the hydrolysis of compound **5**, which comprised amide in molecules by AChE. The resulting methane sulfonamide from compound **5** broke the extra disulfide bond of Ellman’s reagent to produce more TNB. Therefore, it was possible to assume that this substance encouraged an enzymatic activity of AChE. In the case of compound **12**, no adverse outcome was seen. It is probably due to the resonance effect in 4-methylbenzene sulfonamide. It could decrease the nucleophilic property of sulfonamide -NH_2_ of compound **12**. Thus, low TNB from the hydrolysis of disulfide bond of Ellman’s reagent would be produced. 

### 2.3. Molecular Modeling

The Aβ fragments chosen for this experiment must contain the amino acid sequences of the active binding site, which are employed to bind together to create an oligomer and fibril, respectively. As in other investigations, the Aβ monomer sequence was composed of two major parts that were hydrophobic regions around amino acid sequences 17–21 and 29–42 and the hydrophilic portion around sequences 22–28 [11,12,13,14,15]. Those two components were essential for maintaining the formation of the secondary structure by intra- and intermolecular interactions of a salt bridge, ion-dipole between Asp23 and Lys28, and hydrophobic interaction between Leu17-Ala21 and Gly29-Ala42. This region served as the origin of the backbone of oligomers and positioned a long chain of Aβ protein into an appropriate position and then formed intermolecular interaction through H-bonding to produce fibrils and plaques. Consequently, the substance that can interfere with those regions by forming a complex with Aβ protein may inhibit the formation of Aβ plaques. 

**Figure 4:**
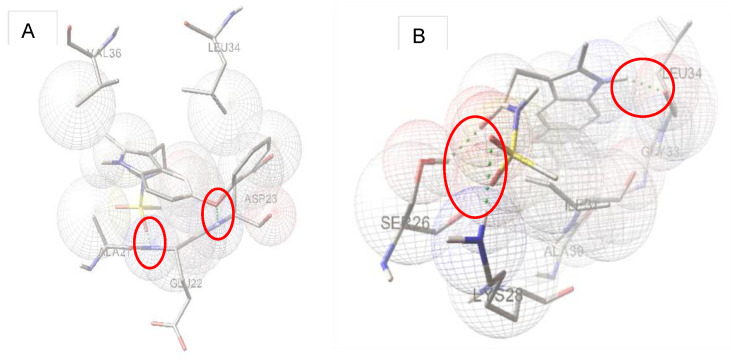
Docking positioning and interaction of compound **5** with (**A**) 2BEG (Aβ_17–42_) and (**B**) 1QWP (Aβ_25–35_); green dots are H-bonds.

The information in Table 6 and Table 7 demonstrated binding energy and interactions of newly developed structures on Aβ_17–42_ (2BEG) and Aβ_25–35_ (1QWP), respectively. The structures interacting with Asp23/Lys28 at the salt bridge region were focused. The most intriguing structures were compounds **3**, **5**, and **11**, which showed interaction with Asp23/Lys28 via H-bonds in both Aβ_17–42_ and Aβ_25–35_. According to the lowest energy shown in Table 7, compound **5** demonstrated a particularly tight binding on Aβ_25–35_. Figure 4 shows a docking position of compound **5** on Aβ_17–42_ and Aβ_25–35_. Compounds **4**, **6**, **7**, **8**, and **12** were also intriguing because the interaction between compounds and salt bridge had been observed in the 2BEG model. Compounds **9** and **10** were also bound to 2BEG and 1QWP. However, the binding was loose and not in a key area.

The docking simulations of compound **5** on the Aβ_17–42_ model (Figure 4A) revealed that the phenoxy moiety formed an H-bond with Asp23, the essential amino acid in the hydrophilic area for salt bridge formation, which led to the development of the Aβ oligomer backbone. Additionally, the sulfonamide of compound **5** also created an H-bond with the critical Glu22 of the hydrophilic area. In the case of the Aβ_25–35_ monomer model (Figure 4B), the sulfonamide of compound **5** might create an H-bond interaction with Lys28, another essential pairing amino acid for salt bridge production. The results of molecular docking tests showed the strongest potency against the aggregation of Aβ protein of compound **5**.

### 2.4. In Silico Prediction of CNS Assess

All phenoxyindole derivatives predicted their brain penetration using ADMET Predictor. Log BB [log (C_brain_/C_blood_)] value was used to predict how effectively the molecules can penetrate the blood–brain barrier (BBB). When Log BB is in the range of 1 to −1, it shows that the substance can permeate the BBB [52]. Table 8 displays the Log BB value of the derivatives. The anticipated outcomes indicate that most phenoxyindole derivatives can cross the BBB. Among three active substances, compounds **3**, **5**, and **8**, compound **8**, with its limiting structural features for brain exposure, exhibits strong passive penetration capacity across the BBB from blood vessels to the CNS. However, compounds **3** and **5** exhibit modest penetration ability.

**Figure 5:**
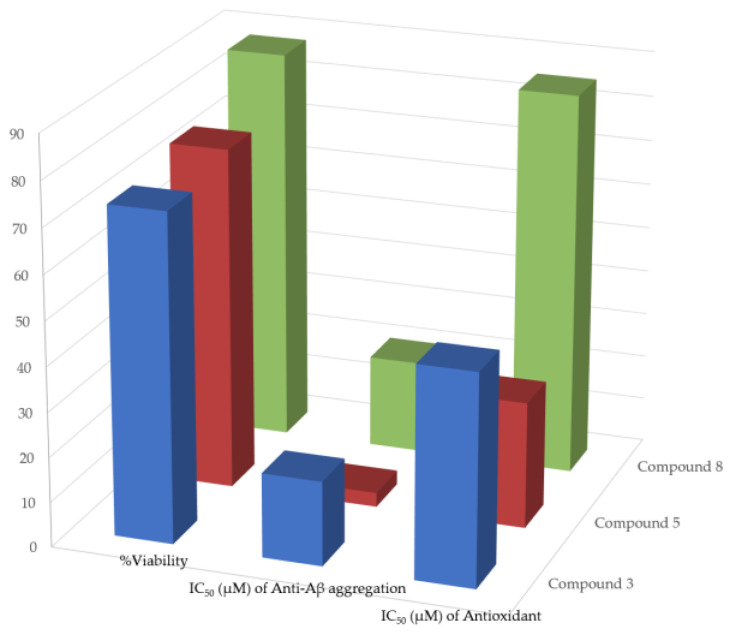
Correlations of %viability of SK-N-SH cells, IC_50_ values (µM) of anti-Aβ aggregation, and IC_50_ values (µM) of antioxidants of compounds **3**, **5**, and **8**.

All developed compounds, except compounds **9** and **10**, could protect SK-N-SH cells at the half-maximal inhibitory concentration of anti-Aβ aggregation, as shown by the biological activities testing. Several compounds, particularly compounds **3**, **4**, **5**, **6**, **7**, **8**, **11**, and **12**, revealed remarkably the relationship between the %viability of SK-N-SH cells and IC_50_ values of anti-Aβ aggregation. Additionally, the antioxidant activity of compounds **3**, **5**, and **8** linked also with both earlier activities. Figure 5 displays the associations of the %viability of SK-N-SH cells, IC_50_ values of anti-Aβ aggregation, and IC_50_ values of antioxidants of compounds **3**, **5**, and **8**. No significant potency of all synthesized compounds against AChE was found. These results suggested that the SK-N-SH neuroblastoma cells might be protected by our proposed phenoxyindole derivatives from Aβ_42_-induced cell death by adding a minor polar functional group to the side chain, such as carboxyl, methylamine, or methanesulfonamide. The particular biological mechanisms of this protection may involve the anti-Aβ aggregation and antioxidant. The most effective design compound in this study, which showed very powerful specific efficacy against Aβ aggregation and free radical generation, was compound **5**. Although the neuroprotective effect of compound **8** against Aβ induced toxicity demonstrated the highest potency with cell viability of 87.90%, its unique mechanisms to strengthen the protective effect may serve other purposes given that it showed low to moderate activities of all biological activities testing in this study. Thus, a thorough biological examination of compound **8** should be carried out in the future. In silico investigation of CNS permeation indicates compound 8 can cross the BBB.

## 3. Conclusions

In conclusion, a number of novel, indole-based scaffolds, phenoxyindole derivatives, were designed and created. Some were shown to have putative anti-Aβ aggregation and antioxidative properties with a strong protective effect of neuronal SK-N-SH neuroblastoma against Aβ_42_-induced cell death at their IC_50_ levels. However, no developed compounds demonstrated this study’s high effect of anticholinesterase. Molecular docking was used to create the phenoxyindole scaffold that contained several alkyl substitutions on 3-position for anti-Aβ aggregation. Structure–activity relationships through molecular modeling discovered that small polar moieties, including methylamine in compound **4** and methanesulfonamide in compound **5,** aid hydrogen bond formation, especially at the salt bridge region in binding to the Aβ peptide. Compound **5** had remarkable properties against Aβ aggregation and radical production. However, the % cell viability of this substance is not connected to the most effective specific mechanisms discovered through in vitro testing. It should be noted that certain aspects of the cellular absorption system may limit the capacity to enter cells of this substance. Therefore, the physicochemical aspects involved in the cell permeability of compound **5** should be further researched. In the case of compound **8**, the neuroprotective effect against Aβ_42_-induced toxicity displayed the most potency. However, its unique mechanisms to enhance the protective impact may serve different purposes given that it demonstrated modest to moderate antioxidant and anti-Aβ aggregation capabilities. More biological mechanisms related to the Alzheimer’s etiology of this substance should be further studied. In silico analysis of CNS permeation revealed a high ability to cross the BBB for compound **8**. According to the findings of this study, it can be concluded that compounds **5** and **8** have become new, potential lead compounds for the new therapeutic intervention against Alzheimer’s disease. Further in vivo testing will be revealed in due course.

## 4. Material and Methods 

All the chemicals were purchased from commercial suppliers and were used without any purification. Acetylcholinesterase from Electrophorus electricus (electric eel) type VI-S was produced from Sigma. Amyloid beta (Aβ_1–42_) was purchased from the American Peptide Company (Sunnyvale, CA, USA). SK-N-SH human neuroblastoma cell line was obtained from the American Type Culture Collection (ATCC HTB-11). The melting point of all compounds was determined on an Electrothermal model 9100 capillary melting point apparatus. Infrared (IR) spectra were run on Fourier Transform Infrared Spectroscopy (FT-IR) (Nicolet 6700, Thermo Scientific, Waltham, MA, USA) using the direct compression technique and record in cm^−1^. Proton and carbon nuclear magnetic resonance (^1^H NMR, ^13^C NMR) spectra were obtained on an Advance 300 MHz NMR Spectroscopy (DPX-3000, Bruker, Switzerland) at 300 MHz and 75.45 MHz, respectively. Chemical shifts (δ) were reported in parts per million (ppm). The NMR solvents used were deuterated dimethylsulfoxide (DMSO-d6, δ = 2.49 ppm) and deuterated chloroform (CDCl_3_, δ = 7.25 ppm). Mass spectra were determined on a LCQ Fleet Ion Trap Mass Spectrometry using the ESI method. Silica gel 60 (Merck, Art 7734) was used for column chromatography as an adsorbent. The ratio of sample and the adsorbent was about 1:40–60 by weight. The mobile phases developed for thin-layer chromatography (TLC) were generally applied for a column. TLC investigations routinely used for chromatographic separations were carried out on silica gel F_254_ coated aluminum sheets 20 × 20 cm (Merck, 0.2 mm thickness). The components were detected by visualization under ultraviolet (UV) light at λ of 254 and 365 nm. All solvents were reagent grade and, when necessary, were purified and dried by standard methods. Starting materials and reagents were obtained from commercial suppliers.

### 4.1. Synthesis

#### 4.1.1. 4-phenoxyhydrazine (**1**) 

4-Phenoxyaniline 1.0 eq (2.0 g, 10.8 mmol) was dissolved in a concentrated aqueous hydrochloric acid (HCl) solution (22 mL) and cooled to 0 °C and then 22 g ice was added into the reaction. To this, a cooling solution of NaNO_2_ 2.0 eq (1.49 g, 21.6 mmol) in water (7.6 mL) was added over 15 min whilst maintaining the temperature below 10 °C. The reaction was then cooled to 0 °C and stirred for 2 h (until the color of the reaction turned to clear light yellow). To the reaction mixture, sulfamic acid 1.0 eq (1.05 g, 10.8 mmol) was added portion-wise over 20 min (it produces gas); a solution of tin (II) chloride dihydrate 4.0 eq (9.78 g, 43.2 mmol) in a concentrated aqueous HCl solution (8.2 mL) was then added dropwise over a 20 min whilst maintaining the temperature below 15 °C. The color of the reaction mixture changed from pale yellow to white when the reaction was complete. The reaction mixture then stirred at 0 °C for 2 h was then basified to pH 14 with 5M NaOH (aq) whilst maintaining the reaction temperature below 30 °C. The reaction mixture was then rapidly extracted with dichloromethane (DCM) (100 mL × 2), the organics were dried over anhydrous Na_2_SO_4_ and solvent was removed in vacuo without heating to give crude product **1** as a pale-yellow solid (2.19 g, 101%—contains trace impurities). It was used as a starting material for the next step without further purification.

#### 4.1.2. 1-Ethylidene-2-(4-phenoxyphenyl) Hydrazine (**2**)

To a clear pale-yellow solution of *p*-phenoxy phenylhydrazine 1.0 eq (200 mg, 1.0 mmol) in 15 mL of dry DCM, acetaldehyde 2 eq (88 mg, 2.0 mmol) was added. The mixture allowed standing for 2 h (the color was changed to light yellow). Then it was poured into 25 mL of water and extracted with DCM (25 mL × 2). The organic phase was combined, dried over anhydrous Na_2_SO_4_, and evaporated under vacuum. The crude imine (**2**) was obtained as a yellow-brown oil (215 mg, 95%). It was transferred to further step without purification.

#### 4.1.3. 2-Methyl-3-(carboxymethyl)-5-phenoxy Indole (**3**) 

1-Ethylidene-2-(4-phenoxyphenyl)hydrazine 1 eq (226 mg, 1.0 mmol) and levulinic acid 1.2 eq (140mg, 1.2 mmol) were dissolved in 2 N acetic acid (10 mL). The resulting solution was stirred at room temperature for 6 h in open-air (the color change from violet to brown-yellow and finally light brown yellow) and then added glacial acetic acid (10 mL). The resulting solution was heated at 80 °C with stirring for 3 h, cooled, diluted with water (200 mL), and then extracted with DCM (25 mL × 4). The combined organic extracts were washed with saturated NaHCO_3_ solution (25 mL × 2) and brine (20 mL), dried over anhydrous Na_2_SO_4_, and then concentrated in vacuo. The crude product was purified by column chromatography. The product was eluted with hexane:ethyl acetate (2:1); after collecting and removing the solvent, 251 mg (89%) of the resulting close ring product (**3**) was obtained as a dark brown solid: m.p. 113–115 °C; TLC *R*_f_ = 0.33 (SiO_2_, EtOAc:Hexane:1% glacial acetic acid/1:1:1); FT-IR (KBr) ν (cm^−1^) 3226, 3053, 2905, 1671 and 1234 cm^−1^; ^1^H NMR (300 MHz), CDCl_3_, δ = 7.88 (s, 1H, indole-H1), 7.24 (t, *J* = 7.54 Hz, 2H, PhO-H3′, PhO-H5′, 7.18 (d, *J* = 2.08 Hz, 1H, indole-H4), 7.16 (d, *J* = 8.66 Hz, 1H, indole-H7), 6.97 (t, *J* = 7.34 Hz, 1H, PhO-H4′), 6.93 (d, *J* = 7.76 Hz, 2H, PhO-H2′, PhO-H6′), 6.84 (dd, *J* = 8.62, 2.23 Hz, 1H, indole-H6), 3.60 (s, 2H, -CH_2_-), 2.32 (s, 3H, -CH_3_); ^13^C NMR (75.45 MHz), CDCl_3_, δ = 165.48, 157.18, 155.72, 154.79, 137.24, 130.54, 127.09, 123.95, 119.01, 118.75, 27.52, 23.21; MS (ESI) *m/z* 282, 236, 189, 144; HRMS calcd for C_17_H_15_NO_3_, *m/z*: 303.1052 [M-H + Na] ^+^; Found, *m/z* 303.1097. 

1-Ethylidene-2-(4-phenoxyphenyl)hydrazine 1 eq (226 mg, 1.0 mmol) and levulinic acid 1.2 eq (140 mg, 1.2 mmol) were dissolved in 2 N acetic acid (10 mL). The resulting solution was stirred at room temperature for 6 h in open-air (the color change from violet to brown-yellow and finally light brown yellow) and then added glacial acetic acid (10 mL). The resulting solution was heated at 80 °C with stirring for 3 h, cooled, diluted with water (200 mL), and then extracted with DCM (25 mL × 4). The combined organic extracts were washed with saturated NaHCO_3_ solution (25 mL × 2) and brine (20 mL), dried over anhydrous Na_2_SO_4_, and then concentrated in vacuo. The crude product was purified by column chromatography. The product was eluted with hexane:ethyl acetate (2:1); after collecting and removing the solvent, 251 mg (89%) of the resulting close ring product (**3**) was obtained as a dark brown solid: m.p. 113–115 °C; TLC *R*_f_ = 0.33 (SiO_2_, EtOAc:Hexane:1% glacial acetic acid/1:1:1); FT-IR (KBr) ν (cm^−1^) 3226, 3053, 2905, 1671 and 1234 cm^−1^; ^1^H NMR (300 MHz), CDCl_3_, δ = 7.88 (s, 1H, indole-H1), 7.24 (t, *J* = 7.54 Hz, 2H, PhO-H3′, PhO-H5′, 7.18 (d, *J* = 2.08 Hz, 1H, indole-H4), 7.16 (d, *J* = 8.66 Hz, 1H, indole-H7), 6.97 (t, *J* = 7.34 Hz, 1H, PhO-H4′), 6.93 (d, *J* = 7.76 Hz, 2H, PhO-H2′, PhO-H6′), 6.84 (dd, *J* = 8.62, 2.23 Hz, 1H, indole-H6), 3.60 (s, 2H, -CH_2_-), 2.32 (s, 3H, -CH_3_); ^13^C NMR (75.45 MHz), CDCl_3_, δ = 165.48, 157.18, 155.72, 154.79, 137.24, 130.54, 127.09, 123.95, 119.01, 118.75, 27.52, 23.21; MS (ESI) *m/z* 282, 236, 189, 144; HRMS calcd for C_17_H_15_NO_3_, *m/z*: 303.1052 [M-H + Na] ^+^; Found, *m/z* 303.1097. 

#### 4.1.4. General Procedure for the Synthesis of 3-Substituted-5-phenoxyindole Derivatives

Compound **3** 1 eq (280 mg, 1.0 mmol), EDCI 1.2 eq (187 mg, 1.2 mmol), and HOBt 1.05 eq (161 mg, 1.05 mmol) were dissolved in DCM (3 mL) as portion 1. Then a solution of portion 2 that consisted of amine or acid derivative 1.05 eq, triethylamine 1.2 eq (122 mg, 1.2 mmol), and DCM (2 mL) was added slowly to portion 1. The reaction mixture was allowed to stir at room temperature for 3 h. The resulting solution was diluted with 2N HCl (5 mL) and then extracted with DCM (10 mL × 2). The organic extracts were combined and washed with saturated NaHCO_3_ solution (5 mL × 2) and brine (5 mL), dried over anhydrous Na_2_SO_4_, and then concentrated in vacuo. The crude product was purified by column chromatography using ethyl acetate:n-hexane as an eluting agent.

#### 4.1.5. 2-Methyl-3-(methyl ethanamide-2-yl)-5-phenoxy Indole (**4**)

Yellow solid; yield = 50%; m.p. 65–67 °C; TLC *R*_f_ 0.22 (SiO_2_, EtOAc:Hexane/5:1); FT-IR (KBr) ν (cm^−1^) 3407, 3276, 1648, 1479 and 1219 cm^−1^; ^1^H NMR (300 MHz), CDCl_3_, δ = 10.88 (s, 1H, indole-H1), 8.31 (S, 1H, CO-NH-), 7.30 (t, *J* = 7.59 Hz, 2H, PhO-H3′, PhO-H5′, 7.25 (d, *J* = 8.94 Hz, 1H, indole-H7), 7.17 (d, *J* = 1.67 Hz, 1H, indole-H4), 7.00 (t, *J* = 7.32 Hz, 1H, PhO-H4′), 6.87 (d, *J* = 7.77 Hz, 2H, PhO-H2′, PhO-H6′), 6.72 (dd, *J* = 8.57, 2.30 Hz, 1H, indole-H6), 3.36 (s, 2H, -CH_2_-), 2.51 (s, 3H, -NH-CH_3_), 2.34 (s, 3H, -CH_3_); ^13^C NMR (75.45 MHz), CDCl_3_, δ = 171.58, 159.76, 148.48, 135.21, 132.62, 130.09, 129.60, 122.09, 116.91, 113.81, 111.69, 109.33, 105.76, 31.95, 26.09, 11.97; MS (ESI) *m/z* 295, 236, 143; HRMS calcd for C_18_H_18_N_2_O_2_, *m/z*: 317.1260 [M + Na]^+^; found, *m/z*: 317.1255. 

#### 4.1.6. 2-Methyl-3-(methylsulfonyl ethanamide-2-yl)-5-phenoxy Indole (**5**)

Yellow-brown solid; yield = 50%; m.p. 175–177 °C; TLC *R*_f_ 0.22 (SiO_2_, EtOAc:Hexane/1:1); FT-IR (KBr) ν (cm^−1^) 3385, 3241, 1695, 1342 and 1219 cm^−1^; ^1^H NMR (300 MHz), CDCl_3_, δ = 11.40 (s, 1H, -NH-SO_2_-), 8.72 (s, 1H, indole-H1), 7.71 (t, *J* = 7.94 Hz, 2H, PhO-H3′, PhO-H5′, 7.68 (d, *J* = 8.52 Hz, 1H, indole-H7), 7.60 (d, *J* = 1.67 Hz, 1H, indole-H4), 7.41 (t, *J* = 7.32 Hz, 1H, PhO-H4′), 7.29 (d, *J* = 7.77 Hz, 2H, PhO-H2′, PhO-H6′), 7.15 (dd, *J* = 8.57, 2.30 Hz, 1H, indole-H6), 3.99 (s, 2H, -CH_2_-), 3.57 (s, 3H, -SO_2_-CH_3_), 2.75 (s, 3H, -CH_3_); ^13^C NMR (75.45 MHZ), CDCl_3_, δ = 171.41, 159.69, 148.69, 135.81, 132.57, 130.13, 129.48, 122.16, 116.95, 114.02, 111.88, 109.17, 103.77, 41.36, 31.93, 11.98; MS (ESI) *m/z* 358, 236; HRMS calcd for C_18_H_18_N_2_O_4_S, *m/z*: 381.0879 [M + Na]^+^; found, *m/z*: 381.0868.

#### 4.1.7. 2-Methyl-3-(ethyl ethanoate-2-yl)-5-phenoxy Indole (**6**)

Yellow solid; yield = 90%; m.p. 79–81 °C; TLC *R*_f_ 0.67 (SiO_2_, EtOAc:Hexane/1:1); FT-IR (KBr) ν (cm^−1^) 3322, 2979, 1712, 1489 and 1241 cm^−1^; ^1^H NMR (300 MHz), CDCl_3_, δ = 10.96 (s, 1H, indole-H1), 7.31 (t, *J* = 7.95 Hz, 2H, PhO-H3′, PhO-H5′, 7.28 (d, *J* = 8.53 Hz, 1H, indole-H7), 7.052 (d, *J* = 2.25 Hz, 1H, indole-H4), 7.049 (t, *J* = 7.35 Hz, 1H, PhO-H4′), 6.88 (d, *J* = 7.75 Hz, 2H, PhO-H2′, PhO-H6′), 6.75 (dd, *J* = 8.59, 2.34 Hz, 1H, indole-H6), 4.00 (q, *J* = 7.11 Hz, 2H, -O-CH_2_-CH_3_), 3.60 (s, 2H, -CH_2_-), 2.33 (s, 3H, -CH_3_), 1.10 (t, *J* = 7.11 Hz, 3H, -O-CH_2_-CH_3_); ^13^C NMR (75.45 MHz), CDCl_3_, δ = 171.92, 159.58, 148.87, 135.31, 132.53, 130.11, 129.37, 122.26, 117.17, 113.93, 111.90, 108.78, 104.11, 60.43, 30.20, 14.53, 11.80; MS (ESI) *m/z* 310, 236; HRMS calcd for C_19_H_19_NO_3_, *m/z*: 332.1257 [M + Na]^+^; found, *m/z*: 332.1253.

#### 4.1.8. 2-Methyl-3-(methyl ethanoate-2-yl)-5-phenoxy Indole (**7**)

Red-brown solid; yield = 90%; m.p. 90–92 °C; TLC *R*_f_ 0.60 (SiO_2_, EtOAc:Hexane/1:1); FT-IR (KBr) ν (cm^−1^) 3319, 3039, 1699, 1481 and 1222 cm^−1^; ^1^H NMR (300 MHz), CDCl_3_, δ = 10.98 (s, 1H, indole-H1), 7.31 (t, *J* = 7.95 Hz, 2H, PhO-H3′, PhO-H5′, 7.27 (d, *J* = 8.53 Hz, 1H, indole-H7), 7.05 (d, *J* = 2.25 Hz, 1H, indole-H4), 7.01 (t, *J* = 7.35 Hz, 1H, PhO-H4′), 6.88 (d, *J* = 7.75 Hz, 2H, PhO-H2′, PhO-H6′), 6.74 (dd, *J* = 8.59, 2.34 Hz, 1H, indole-H6), 3.63 (s, 2H, -CH_2_-), 3.55 (s, 3H, -O-CH_3_), 2.32 (s, 3H, -CH_3_); ^13^C NMR (75.45 MHz), CDCl_3_, δ = 172.42, 159.58, 148.83, 135.35, 132.53, 130.12, 129.38, 122.25, 117.13, 113.91, 108.78, 104.00, 51.92, 29.90, 11.79; MS (ESI) *m/z* 296, 236, 143; HRMS calcd for C_18_H_17_NO_3_, *m/z*: 318.1100 [M + Na]^+^; found, *m/z*: 318.1088.

#### 4.1.9. 2-Methyl-3-(cyano ethanamide-2-yl)-5-phenoxy Indole (**8**)

Red-brown solid; yield = 57%; m.p. > 210 °C; TLC *R*_f_ 0.40 (SiO_2_, EtOAc:Hexane:1% glacial acetic acid/1); FT-IR (KBr) ν (cm^−1^) 3246, 2378, 1676, 1555 and 1243 cm^−1^; ^1^H NMR (300 MHz), CDCl_3_, δ = 7.66 (s, 1H, indole-H1), 7.65 (s, 1H, -CO-NH-CN), 7.43 (t, *J* = 8.38 Hz, 1H, indole-H7), 7.28 (t, *J* = 7.97 Hz, 2H, PhO-H3′, PhO-H5′), 7.04 (t, *J* = 7.39 Hz, 1H, PhO-H4′) 6.95 (d, *J* = 2.37 Hz, 1H, indole-H4), 6.91 (dd, *J* = 12.17, 2.51 Hz, 2H, PhO-H2′, PhO-H6′), 6.90 (dd, *J* = 11.02, 2.16 Hz, 1H, indole-H6), 3.24 (s, 2H, -CH_2_-), 2.18 (s, 3H, -CH_3_); ^13^C NMR (75.45 MHz), CDCl_3_, δ = 178.34, 157.08, 155.09, 152.81, 151.26, 141.05, 129.05, 128.34, 123.57, 121.04, 119.33, 118.67, 112.93, 66.80, 49.58, 15.35; MS (ESI) *m/z* 306, 236. HRMS calcd for C_18_H_15_N_3_O_2_, *m/z*: 328.105647 [M + Na]^+^; yound, *m/z*: 328.1052.

#### 4.1.10. 2-Methyl-3-(4-methylbenzyl ethanamide-2-yl)-5-phenoxy Indole (**9**)

White solid; yield = 72%; m.p. 130–132 °C; TLC *R*_f_ 0.50 (SiO_2_, EtOAc:Hexane/1:1); FT-IR (KBr) ν (cm^−1^) 3382, 3269, 2918, 1645 and 1223 cm^−1^; ^1^H NMR (300 MHz), CDCl_3_, δ = 10.88 (s, 1H, indole-H1), 8.29 (t, *J* = 5.6 Hz, 1H, -CO-NH-tolyl), 7.28 (t, *J* = 7.64 Hz, 2H, PhO-H3′, PhO-H5′), 7.25 (t, *J* = 8.38 Hz, 1H, indole-H7), 7.24 (d, *J* = 2.88 Hz, 1H, indole-H4), 7.06 (d, *J* = 7.52 Hz, 2H, tolyl-H2′, tolyl-H6′), 7.03 (t, *J* = 7.62 Hz, 1H, PhO-H4′), 6.98 (d, *J* = 8.08 Hz, 1H, PhO-H2′, PhO-H6′), 6.86 (d, *J* = 8.08 Hz, 2H, tolyl-H3′, tolyl-H5′), 6.72 (t, *J* = 8.47 Hz, 1H, indole-H6), 4.16 (d, *J* = 5.72 Hz, -NH-CH_2_-tolyl), 3.44 (s, 2H, -CH_2_-), 2.35 (s, 3H, -CH_3_), 2.21 (s, 3H, Ph-CH_3_); ^13^C NMR (75.45 MHz), CDCl_3_, δ = 171.39, 160.18, 148.62, 137.25, 136.36, 132.94, 130.36, 129.91, 127.80, 114.23, 111.98, 109.84, 106.13, 42.62, 32.27, 21.36; MS (ESI) *m/z* 384, 279, 236. HRMS calcd for C_25_H_24_N_2_O_2_, *m/z*: 407.1729 [M + Na]^+^; found, *m/z*: 407.1729.

#### 4.1.11. 2-Methyl-3-(4-fluorobenzyl ethanamide-2-yl)-5-phenoxy Indole (**10**)

Yellow solid; yield = 66%; m.p. 130–132 °C; TLC *R*_f_ 0.45 (SiO_2_, EtOAc:Hexane/1:1); FT-IR (KBr) ν (cm^−1^) 3394, 3267, 1650, 1483 and 1223 cm^−1^; ^1^H NMR (300 MHz), CDCl_3_, δ = 10.90 (s, 1H, indole-H1), 8.37 (t, *J* = 5.82 Hz, 1H, -CO-NH-PhF), 7.32 (t, *J* = 7.96 Hz, 2H, PhO-H3′, PhO-H5′), 7.27 (d, *J* = 8.47 Hz, 1H, indole-H7), 7.21 (d, *J* = 2.28 Hz, 1H, indole-H4), 7.19 (d, *J* = 5.70 Hz, 2H, PhF-H3′, PhF-H5′), 7.01 (d, *J* = 5.85 Hz, 2H, PhF-H2′, PhF-H6′), 7.01 (t, *J* = 8.94 Hz, 1H, PhO-H4′), 6.86 (d, *J* = 8.21 Hz, 2H, PhO-H2′, PhO-H6′), 6.73 (dd, *J* = 8.56, 2.25 Hz, 1H, indole-H6), 4.19 (d, *J* = 5.74 Hz, -NH-CH_2_-PhF), 3.45 (s, 2H, -CH_2_-), 2.35 (s, 3H, -CH_3_); ^13^C NMR (75.45 MHz), CDCl_3_, δ = 171.22, 159.87, 148.62, 148.39, 136.24, 135.29, 129.56, 122.03, 115.42, 115.14, 113.94, 111.72, 109.48, 105.74, 41.92, 29.18, 11.99; MS (ESI) *m/z* 388, 236. HRMS calcd for C_24_H_21_FN_2_O_2_, *m/z*: 411.1479 [M + Na]^+^; found, *m/z*: 411.1472.

#### 4.1.12. 2-Methyl-3-(phenyl ethanamide-2-yl)-5-phenoxy Indole (**11**) 

Pale-yellow solid; yield = 65%; m.p. 148–151 °C; TLC *R*_f_ 0.44 (SiO_2_, EtOAc:Hexane/1:1); FT-IR (KBr) ν (cm^−1^) 3410, 3251, 1664, 1477 and 1218 cm^−1^; ^1^H NMR (300 MHz), CDCl_3_, δ = 8.55 (s, 1H, indole-H1), 7.47 (s,1H, -CO-NH-Ph), 7.31 (d, *J* = 8.19 Hz, 2H, Ph-H2′, Ph-H6′), 7.239 (t, *J* = 7.77 Hz, 2H, Ph-H3′, Ph-H5′), 7.238 (d, *J* = 8.78 Hz, 1H, indole-H7), 7.22 (t, *J* = 7.98 Hz, PhO-H3′, PhO-H5′), 7.16 (d, *J* = 1.92 Hz, 1H, indole-H4), 7.01 (t, *J* = 7.39 Hz, 1H, Ph-H4′), 6.98 (t, *J* = 7.36 Hz, 1H, PhO-H4′), 6.92 (d, *J* = 7.60 Hz, 2H, PhO-H2′, PhO-H6′), 6.90 (dd, *J* = 8.56, 2.20 Hz, 1H, indole-H6), 3.73 (s, 2H, -CH_2_-), 2.37 (s, 3H, -CH_3_); ^13^C NMR (75.45 MHz), CDCl_3_, δ = 170.15, 148.59, 139.68, 135.41, 132.60, 129.63, 129.08, 122.10, 116.93, 113.85, 111.76, 109.34, 105.54, 49.05, 12.07; MS (ESI) *m/z* 356, 236. HRMS calcd for C_23_H_20_N_2_O_2_, *m/z*: 379.1416 [M + Na]^+^; found, *m/z*: 379.1417.

#### 4.1.13. 2-Methyl-3-(4-methylbenzesulfonyl ethanamide-2-yl)-5-phenoxy Indole (**12**)

Pale-brown solid; yield = 70%; m.p. 175–177 °C; TLC *R*_f_ 0.36 (SiO_2_, EtOAc:Hexane/1:1); FT-IR (KBr) ν (cm^−1^) 3426, 3270, 1710, 1377 and 1222 cm^−1^; ^1^H NMR (300 MHz), CDCl_3_, δ = 12.13 (brs,1H, -CO-NH-SO_2_), 10.90 (s, 1H, indole-H1), 7.74 (d, *J* = 8.18 Hz, 2H, tolyl-H2′, tolyl-H6′), 7.33 (t, *J* = 10.86 Hz, 2H, PhO-H3′, PhO-H5′), 7.32 (d, *J* = 8.24 Hz, 1H, indole-H7), 7.28 (t, *J* = 10.17 Hz, PhO-H4′), 7.23 (d, *J* = 10.71 Hz, 2H, PhO-H2′, PhO-H6′), 7.09 (d, *J* = 1.39 Hz, 1H, indole-H4), 6.86 (d, *J* = 8.30 Hz, 2H, tolyl-H3′, tolyl-H5′), 6.71 (dd, *J* = 8.54, 1.81 Hz, 1H, indole-H6), 3.51 (s, 2H, -CH_2_-), 2.33 (s, 3H, Ph-CH_3_), 2.26 (s, 3H, -CH_3_); ^13^C NMR (75.45 MHz), CDCl_3_, δ = 170.33, 148.53, 144.58, 135.70, 132.51, 129.89, 129.76, 122.12, 116.83, 114.02, 111.83, 109.15, 103.71, 31.84, 11.86; MS (ESI) *m/z* 434, 236. HRMS calcd for C_24_H_22_N_2_O_4_S, *m/z*: 457.1192 [M + Na]^+^; found, *m/z*: 457.1199.

### 4.2. Biological Assay

#### 4.2.1. Evaluation of Antiaggregation Activity (Thioflavin T Fluorescence Assay: ThT Assay) 

Aβ_1–42_ salt was dissolved in 10 mM sodium hydroxide and diluted in 50 mM Tris-HCl (pH 7.4) to obtain a stock solution at 25 µM. Stock solutions of synthesized compounds and positive control (curcumin) were prepared in DMSO. Each compound was diluted in 50 mM Tris-HCl (pH 7.4) to achieve a concentration between 10 nM and 100 µM using 5 concentration points. A total of 1 µL of each sample solution was added to 96-well plates. Each concentration was prepared in independent triplicate and a reagent blank was included; 9 µL of 25 µM stock solution Aβ_1–42_ is added to each well. The plate was incubated in the dark at room temperature for 24 h with no agitation. After an incubation period, 200 µL of 5 µM ThT in 50 mM Tris-HCl (pH 7.4) was added to each well. Fluorescence was measured on a SpectraMax GEMINI EM (Molecular Devices, Sunnyvale, CA, USA) dual scanning microplate spectrofluorometer with excitation and emission wavelengths at 446 nm and 500 nm, respectively [53]. The percent of antiaggregation was calculated as follows: [1 − (F_sample_ − F_blank_)/F_control_] × 100.

#### 4.2.2. Protection of Neuronal SK-N-SH Neuroblastoma against Aβ_42_ Induced Cell Death

Quantitative measurement of cell viability was performed by MTT assay. Briefly, SK-N-SH cells were seeded at a density of 5 × 10^4^ cells/100 µL into each well of 96-well plate and incubated at 37 °C in 5% CO_2_ for 24 h. After incubation, the tested compounds were added at different concentrations, from 0 to 1000 µM. After incubation for 24 h, 50 µL of 3-(4,5-dimethylthiazol-2-yl)-2,5-diphenyltetrazolium bromide (MTT) stock solution (1 mg/mL) was added to each well and incubated at 37 °C for 3 h. Absorbance (A) of each well was measured using a plate reader at a wavelength of 590 nm. Three independent experiments were performed, and results were expressed as mean ± SD. For testing the protective effect, SK-N-SH cells were pretreated with the corresponding half-maximal inhibitory concentrations of tested compounds and positive control curcumin for 3 h. Each concentration was prepared in triplicate. Then, 25 µM of Aβ_1–42_ preincubating in 50 mM Tris-HCl (pH 7.4) at 37 °C for 3 days was added into each well. After incubation for 24 h, 50 µL of MTT stock solution (1 mg/mL) was added to each well and incubated at 37 °C for 3 h. The absorbance (A) of each well was measured using a plate reader at a wavelength of 590 nm. Three independent experiments were performed, and results were expressed as mean ± SD.

The percent of cell viability was calculated as follow: (A_590nm_ of treated cells/A_590nm_ of untreated cells) × 100.

#### 4.2.3. Determination of Morphological Cell Changes

To determine the effect of designed compounds on cell morphology, the observation of cells on phase contrast inverted microscope was performed. SK-N-SH cells were seeded at a density of 2 × 10^5^ cells/well on a 24-well plate and incubated in a CO_2_ incubator at 37 °C for 24 h. After incubation, the medium was removed and replaced with various concentrations of the designed compound and incubated for 24 h. After cells were treated with compounds or Aβ_1–42_, media were removed and treated cells were washed once with phosphate buffer saline (PBS). Then, cells were observed under phase contrast inverted microscope at 400 magnifications.

#### 4.2.4. Evaluation of Acetylcholinesterase Inhibitory Activity

Acetylcholinesterase (AChE) inhibitory activity was assayed by modified Ellman’s method using 96-well microplates. Briefly, for evaluation acetylcholinesterase activity, 125 µL of 3 mM 5,5′-dithiobis(2-nitrobenzoic acid) (DTNB) in 50 mM Tris-HCl buffer pH 8.0 containing 0.1 M NaCl and 0.02 M MgCl_2_·2H_2_O (Buffer III), 25 µL of 15 mM acetylthiocholine iodide (ATCI) in deionized water, 50 µL of 50 mM Tris-HCl buffer pH 8.0 containing 0.1% bovine serum albumin (BSA) (Buffer II), and 25 µL of the tested compound and positive control, galantamine in 50 mM Tris-HCl buffer containing 10% methanol (Buffer I) were added to the wells. The absorbance of the mixture in each well was measured at 405 nm by using a microplate reader. After adding 25 µL of 0.22 U/mL AChE in Buffer II into each well, the measurement of absorbance was carried out again at 405 nm and repeated every 45 s for 10 min cycles by a microplate reader (Infinite M200, Tecan, Switzerland). Inhibitory activity was calculated from the difference between absorbance values of control and sample and resulted in percent of acetylcholinesterase inhibition. The assay was performed in triplicate. The concentration that inhibited 50% of AChE activity (IC_50_) was obtained from the graph that plots between the percentage of acetylcholinesterase inhibition and the concentration of the test compounds. The percent of enzyme inhibition was calculated as follows: {[(A-B) − (C − D)]/(A − B)} × 100, where A and C were the absorbances of the control treatment and test compounds; and B and D were blank of control and test compounds.

#### 4.2.5. Evaluation of Antioxidant Assay

Antioxidant activity of tested compounds was determined by the reaction of stable DPPH radical with some modification. Briefly, various concentrations of test compounds were added in 96-well plates and then the methanolic DPPH solution (0.4 mM, 0.1 mL) into each well. The reaction mixture was placed for 30 min in darkness. The degree of purple DPPH decolorization to yellow DPPH indicated the scavenging capacity of the test compounds. The absorbance of the mixture was determined at 517 nm using a microplate reader and L-ascorbic acid served as a positive control. The percent of antioxidant activity was calculated as follow: [1 − (A_sample_ − A_blank_)/A_control_] × 100.

### 4.3. Docking Studies

The docking calculations were performed using AutoDock program version 4.0 (The Scripps Research Institute, San Diego, CA, USA). The docking study was carried out using the Lamarckian genetic algorithm, applying a standard protocol, with an initial population of 150 randomly placed individuals, a mutation rate of 0.02, and a crossover rate of 0.80. One hundred independent docking runs were carried out for each ligand. Results differing by less than 2.0 Å in position root-mean-square deviation (RMSD) were clustered together and represented by the result with the most favorable free energy of binding.

Ligand preparation: The molecular structures of all compounds were modeled with SYBYL 8.1 molecular modeling program (Tripos Associates, Saint Louis, MI, USA) on an Indigo Elan workstation (Silicon Graphics Inc., Mountain View, CA, USA) using the sketch approach. Each structure was energy minimized using the standard Tripos force field (Powell method and 0.05 kcal/mol Å energy gradient convergence criteria) and the electrostatic charge was assigned by the Gasteiger–Hückel method. These conformations were used as starting conformations to perform docking.

Receptor preparation: The crystal structures of 2BEG and 1QWP complexed with inhibitor were obtained from the Brookhaven Protein Database (PDB). The inhibitor structures and all heteroatoms were removed from the complex structures and added polar hydrogen parameter. Gasteiger charges were assigned.

Grid setup: The grid maps representing the protein in the actual docking process were calculated with AutoGrid. The grids (one for each atom type in the ligand plus one for electrostatic interactions) were chosen to be sufficiently large to include not only the active site, but also significant portions of the surrounding surface. The parameters used for AutoGrid were shown in Table 9.

### 4.4. In Silico Prediction of CNS Access 

ADMET Predictor ™ was used to predict the possibility of phenoxyindole derivatives in blood–brain barrier (BBB) penetration. Molecular structures of phenoxyindole derivatives were drawn two-dimensionally via MedChem Designer ™ and analyzed via ADMET Predictor ™ (version 10.3, Simulation Plus, Lancaster, CA, USA). The section covered the ADMET Predictor models relevant to two separated events, which were predicting the compounds’ ability to penetrate BBB (BBB_Filter) and predicting their retention in the brain once taken up (logBB). These parameters made it possible to anticipate whether a given compound was likely to be active in the central nervous system (CNS). Data used to build the models came from in vivo rat studies. The data for BBB_Filter consisting of 2276 compounds covering a wide range of chemical space were from multiple sources. Partition coefficient values were available for a subset of 462 compounds. It was found that several compounds that penetrated the BBB were pumped back out by efflux transporters, such as P-glycoprotein. The results were consistent with those from the model built by Crivori et al. [52].

## Figures and Tables

**Table 1:** Antiaggregation action of synthesized compounds on Aβ_42_ shown as IC_50_.

Entry	IC_50_ (µM) ± RSD
3	18.81 ± 1.26
4	3.26 ± 1.11
5	3.18 ± 0.87
6	19.14 ± 2.13
7	13.98 ± 1.55
8	20.62 ± 2.79
9	53.67 ± 1.53
10	72.67 ± 2.34
11	13.08 ± 3.11
12	9.48 ± 1.25
Curcumin	11.09 ± 1.78

**Table 2:** The highest concentration of tested compounds that showed nontoxicity on SK-N-SH neuroblastoma cells.

Entry	Concentration (µM)	% Viability ± RSD
3	31.25	90.42 ± 3.69
4	31.25	93.69 ± 3.23
5	62.50	93.04 ± 1.02
6	31.25.	89.47 ± 2.11
7	31.25	93.43 ± 2.15
8	62.50	93.80 ± 3.66
9	31.25	95.21 ± 3.00
10	31.25	80.51 ± 3.96
11	15.63	94.32 ± 3.31
12	15.63	90.73 ± 4.64
Curcumin	15.63	94.36 ± 1.83
DMSO	125 (1.25%)	92.08 ± 2.86

**Table 3:** Neuroprotective effect on SK-N-SH neuroblastoma cells of test compounds at the IC_50_ value, except compounds **9** and **10**, against Aβ induced damage.

Entry	Concentration (µM)	% Viability ± RSD
3	18.81	73.10 ± 0.75
4	3.26	72.27 ± 1.85
5	3.18	76.28 ± 2.39
6	19.14	80.44 ± 4.51
7	13.98	83.74 ± 2.21
8	20.62	87.90 ± 3.26
9	31.25 ^[a]^	80.38 ± 3.54
10	31.25 ^[a]^	63.05 ± 2.70
11	13.08	76.99 ± 1.97
12	9.48	71.85 ± 1.82
Curcumin	11.09	81.37 ± 1.98
Aβ_(1–42)_		51.82 ± 2.19

^[a]^ The concentrations used for compound **9** and **10.**

**Table 4:** Antioxidative activity of synthesized compounds shown as the IC_50_.

Entry	IC_50_ (µM) ± RSD
3	47.12 ± 0.55
4	175.74 ± 1.23
5	28.18 ± 1.40
6	190.43 ± 0.55
7	144.93 ± 1.22
8	85.51 ± 1.60
9	264.78 ± 0.34
10	85.97 ± 0.80
11	111.07 ± 1.31
12	121.19 ± 1.80
Vitamin C	13.35 ± 1.52

**Table 5:** Effect of the synthesized compounds acting as acetylcholinesterase inhibitors at 100 µM.

Entry	% Inhibition ± RSD
3	29.30 ± 4.89
4	9.73 ± 4.49
5	−5.66 ± 2.79
6	19.85 ± 4.96
7	11.02 ± 4.88
8	6.61 ± 4.95
9	12.5 ± 4.57
10	12.45 ± 4.98
11	30.04 ± 4.63
12	31.52 ± 4.90
Galantamine	97.74 ± 4.03

**Table 6:** Binding energy and interaction of new designed structure with 2BEG.

Entry	HP ^[a]^	BE ^[b]^ (kcal/mole)	HA ^[c]^	HD ^[d]^	Cluster
**3**	Ala21, Asp23, Leu34, Met35	−7.37	CO:Asp23	No	13
**4**	Ala21-Asp23, Leu34	−7.60	PhO:Asp23	No	15
**5**	Ala21-Asp23, Leu34, Val36	−7.17	SO_2_:Glu22 PhO:Asp23	No	16
**6**	Ala21-Asp23, Leu34	−6.99	CO:Asp23	No	20
**7**	Ala21-Asp23, Gly33, Leu34	−7.63	CO:Asp23	No	20
**8**	Ala21-Asp23, Gly33-Met35	−7.80	NH(3):Asp23	No	11
**9**	Phe19, Leu34, Ala21-Asp23, Val36-Gly38	−7.24	No	No	15
**10**	Phe19-Asp23, Leu34, Val36	−6.49	No	NH(3):Ala21	12
**11**	Ala21-Asp23, Leu34, Val36	−7.50	CO:Asp23	No	13
**12**	Phe19-Asp23, Leu34, Val36, Gly37	−8.3	SO_2_:Ala21	No	60

^[a]^ HP = hydrophobicity; ^[b]^ BE = binding energy; ^[c]^ HA = hydrogen bond acceptor; ^[d]^ HD = hydrogen bond donor.

**Table 7:** Binding energy and interaction of new designed structure with 1QWP.

Entry	HP ^[a]^	BE ^[b]^ (kcal/mole)	HA ^[c]^	HD ^[d]^	Cluster
**3**	Ser26, Lys28, Ile31, Leu34	−7.79	OH:Lys28	No	60
**4**	Ser26, Asn27, Ala30, Ile31, Gly33, Leu34	−7.35	CO:Ser26, Asn27	No	37
**5**	Ser26, Lys28, Ala30, Ile31, Gly33, Leu34	−8.47	CO:Ser26, SO_2_:Lys28	NH(1): Leu34	54
**6**	Ser26, Asn27, Ala30, Ile31, Gly33, Leu34	−6.93	EtO:Asn27	No	30
**7**	Ser26, Asn27, Ala30, Ile31, Gly33, Leu34	−7.29	CO:Ser26 CH_3_O:Asn27	NH(1): Ile31	41
**8**	Ser26-Lys28, Ile31, Gly33, Leu34	−7.95	CO:Ser26	NH(1): Ile31	74
**9**	Gly25-Asn27, Ala30, Ile31, Gly33, Leu34	−7.14	CO:Asn27	No	32
**10**	Ser26-Lys28, Ile31, Gly33, Leu34	−6.85	CO:Asn27	NH(1): Ile31	36
**11**	Lys28, Ala30, Ile31, Gly33-Met35	−7.65	CO:Lys28	No	30
**12**	Ser26, Lys28, Ala30, Ile31, Gly33-Met35	−7.55	CO:Ser26, SO_2_:Lys28	NH(1): Leu34	20

^[a]^ HP = hydrophobicity; ^[b]^ BE = binding energy; ^[c]^ HA = hydrogen bond acceptor; ^[d]^ HD = hydrogen bond donor.

**Table 8:** Log BB value of phenoxyindole derivatives.

Entry	Log BB [Log (C_brain_/C_blood_)]
**3**	−1.247
**4**	−0.521
**5**	−1.538
**6**	−0.407
**7**	−0.407
**8**	−0.98
**9**	−0.639
**10**	−0.406
**11**	−0.493
**12**	−1.387
**Donepezil**	0.59
**Rivastigmine**	0.401

**Table 9:** AutoGrid parameters.

	Parameters	
PDB code	2BEG	1QWP
Resolution	2.00	2.00
No. Gridpoint in x,y,z	128, 70, 50	48, 48, 48
Spacing (Å)	0.375	0.375
Grid center	Center on macromolecule	Center on macromolecule
Smooth	0.5	0.5

## Data Availability

The data is contained within the article.

## References

[1] Wisniewski T., Ghiso J., Frangione B. (1997). Biology of Aβ amyloid in Alzheimer’s disease. Neurobiol. Dis..

[2] Selkoe D.J. (1991). The molecular pathology of Alzheimer’s disease. Neuron.

[3] Froelich-Fabre S., Bhat R.V. (2004). Mechanisms of tauopathies. Drug Discov. Today Dis. Mech..

[4] Schmidt M., Sachse C., Richter W., Xu C., Faendrich M., Grigorieff N. (2009). Comparison of Alzheimer Aβ(1–40) and Aβ(1–42) amyloid fibrils reveals similar protofilament structures. Proc. Natl. Acad. Sci. USA.

[5] Hardy J., Selkoe D.J. (2002). The amyloid hypothesis of Alzheimer’s disease: Progress and problems on the road to therapeutics. Science.

[6] Haass C. (2004). Take five—BACE and the γ-secretase quartet conduct Alzheimer’s amyloid β-peptide generation. EMBO J..

[7] Tarek M., Arash S., Praveen P.N.R. (2016). Amyloid cascade in Alzheimer’s disease: Recent advances in medicinal chemistry. Eur. J. Med. Chem..

[8] Lichtenthaler S.F. (2012). Alpha-Secretase cleavage of the amyloid precursor protein: Proteolysis regulated by signaling pathways and protein trafficking. Curr. Alzheimer Res..

[9] Lidia P., Celia F. (2019). Therapeutic strategies targeting Amyloid-β in Alzheimer’s disease. Curr. Alzheimer Res..

[10] Haass C., Steiner H. (2002). Alzheimer disease gamma-secretase: A complex story of GxGD-type presenilin proteases. Trends Cell Biol..

[11] Petkova A.T., Buntkowsky G., Dyda F., Leapman R.D., Yau W.M., Tycko R. (2004). Solid State NMR Reveals a pH-dependent Antiparallel β-Sheet Registry in Fibrils Formed by a β-Amyloid Peptide. J. Mol. Biol..

[12] Lührs T., Ritter C., Adrian M., Riek-Loher D., Bohrmann B., Dőbeli H., Schubert D., Riek R. (2005). 3D structure of Alzheimer’s amyloid-β(1–42) fibrils. Proc. Natl. Acad. Sci. USA.

[13] Sciarretta K.L., Gordon D.J., Petkova A.T., Tycko R., Meredith S.C. (2005). Abeta40-Lactam(D23/K28) models a conformation highly favorable for nucleation of amyloid. Biochemistry.

[14] Sato T., Kienlen-Campard P., Ahmed M., Liu W., Li H., Elliott J.I., Aimoto S., Constantinescu S.N., Octave J.N., Smith S.O. (2006). Inhibitors of amyloid toxicity based on β-sheet packing of Aβ40 and Aβ42. Biochemistry.

[15] Shewmaker F., McGlinchey R.P., Wickner R.B. (2011). Structural insights into functional and pathological amyloid. J. Biol. Chem..

[16] Hampel H., Vassar R., De Strooper B., Hardy J., Willem M., Singh N., Zhou J., Yan R., Vanmechelen E., De Vos A. (2021). The β-secretase BACE1 in Alzheimer’s disease. Biol. Psychiatry.

[17] Moussa C.E.H. (2017). Beta-secretase inhibitors in phase I and phase II clinical trials for Alzheimer’s disease. Expert Opin. Investig. Drug.

[18] Ghosh A.K., Tang J. (2015). Prospects of β-Secretase Inhibitors for the Treatment of Alzheimer’s Disease. ChemMedChem.

[19] Polgar T., Keseru G.M. (2014). Structure-based β-secretase (BACE1) inhibitors. Curr. Pharm. Design.

[20] Mekala S., Nelson G., Li Y.-M. (2020). Recent developments of small molecule γ-secretase modulators for Alzheimer’s disease. RSC Med. Chem..

[21] Tang M., Taghibiglou C. (2017). The Mechanisms of action of curcumin in Alzheimer’s disease. J. Alzheimers Dis..

[22] Gagliardi S., Franco V., Sorrentino S., Zucca S., Pandini C., Rota S., Bernuzzi S., Costa A., Sinforiani E., Pansarasa O. (2018). Curcumin and novel synthetic analogs in cell-based studies of Alzheimer’s disease. Front. Pharmacol..

[23] Voulgaropoulou S.D., van Amelsvoort T.A.M.J., Prickaerts J., Vingerhoets C. (2019). The effect of curcumin on cognition in Alzheimer’s disease and healthy aging: A systematic review of pre-clinical and clinical studies. Brain Res..

[24] Murakami K., Irie K. (2019). Three structural features of functional food components and herbal medicine with amyloid β42 anti-aggregation properties. Molecules.

[25] Henriquez G., Gomez A., Guerrero E., Narayan M. (2020). Potential Role of Natural Polyphenols against Protein Aggregation Toxicity: In Vitro, In Vivo, and Clinical Studies. ACS Chem. Neurosci..

[26] Momtaz S., Hassani S., Khan F., Ziaee M., Abdollahi M. (2018). Cinnamon, a promising prospect towards Alzheimer’s disease. Pharmacol. Res..

[27] Rajasekhar K., Chakrabarti M., Govindaraju T. (2015). Function and toxicity of amyloid beta and recent therapeutic interventions targeting amyloid beta in Alzheimer’s disease. Chem. Commun..

[28] Abbas B., Syed N., Jantan I. (2015). Synthetic curcumin analogs as inhibitors of β -amyloid peptide aggregation: Potential therapeutic and diagnostic agents for Alzheimer’s disease. Mini-Rev. Med. Chem..

[29] Mao F., Yan J., Li J., Jia X., Miao H., Sun Y., Huang L., Li X. (2014). New multi-target-directed small molecules against Alzheimer’s disease: A combination of resveratrol and clioquinol. Org. Biomol. Chem..

[30] Alaya S., Genevaux P., Hureau C., Faller P. (2019). (Bio)chemical Strategies To Modulate Amyloid-β Self-Assembly. ACS Chem. Neurosci..

[31] Doig A.J., Derreumaux P. (2015). Inhibition of protein aggregation and amyloid formation by small molecules. Curr. Opin. Struct. Biol..

[32] Mishra P., Ayyannan S.R., Panda G. (2015). Perspectives on Inhibiting β-Amyloid Aggregation through Structure-Based Drug Design. ChemMedChem.

[33] Nie Q., Du X.-Q., Geng M.-Y. (2011). Small molecule inhibitors of amyloid β peptide aggregation as a potential therapeutic strategy for Alzheimer’s disease. Acta Pharmacol. Sin..

[34] Goyal D., Kaur A., Goyal B. (2018). Benzofuran and Indole: Promising scaffolds for drug development in Alzheimer’s disease. ChemMedChem.

[35] Prade E., Bittner H.J., Sarkar R., Lopez del Amo J.M., Althoff-Ospelt G., Multhaup G., Hildebrand P.W., Reif B. (2016). Sulindac sulfide induces the formation of large oligomeric aggregates of the Alzheimer’s disease amyloid-β peptide which exhibit reduced neurotoxicity. Biochemistry.

[36] Shukla M., Govitrapong P., Boontem P., Reiter R.J., Satayavivad J. (2017). Mechanisms of melatonin in alleviating Alzheimer’s disease. Curr. Neuropharmacol..

[37] Bartolini M., Bertucci C., Cavrini V., Andrisano V. (2003). beta-Amyloid aggregation induced by human acetylcholinesterase: Inhibition studies. Biochem. Pharmacol..

[38] Cheignon C., Tomas M., Bonnefont-Rousselot D., Faller P., Hureau C., Collin F. (2018). Oxidative stress and the amyloid beta peptide in Alzheimer’s disease. Redox Biol..

[39] Simunkova M., Alwasel S.H., Alhazza I.M., Jomova K., Kollar V., Rusko M., Valko M. (2019). Management of oxidative stress and other pathologies in Alzheimer’s disease. Arch. Toxicol..

[40] Savelieff M.G., DeToma A.S., Derrick J.S., Lim M.H. (2014). The ongoing search for small molecules to study metal-associated amyloid-β species in Alzheimer’s disease. Acc. Chem. Res..

[41] Robinson B. (1963). The Fischer Indole Synthesis. Chem. Rev..

[42] Przheval’skii N.M., Kostromina L.Y., Grandberg I.I. (1988). New data on the mechanism of the Fischer indole synthesis (review). Chem. Heterocyc. Compd..

[43] Bajwa G., Brown R.K. (1970). Fischer indole synthesis. The reaction of *N*′-methyl-2,6-dimethylphenylhydrazine hydrochloride with 2-methylcyclohexanone and 2,6-dimethylcyclohexanone. Can. J. Chem..

[44] Wolfe L.S., Calabrese M.F., Nath A., Blaho D.V., Miranker A.D., Xiong Y. (2010). Protein-induced photophysical changes to the amyloid indicator dye thioflavin T. Proc. Natl. Acad. Sci. USA.

[45] Hudson S.A., Ecroyd H., Kee T.W., Carver J.A. (2009). The thioflavin T fluorescence assay for amyloid fibril detection can be biased by the presence of exogenous compounds. FEBS J..

[46] Mohamed T., Shakeri A., Tin G., Rao P.N.N. (2016). Structure–activity relationship studies of isomeric 2,4-diaminoquinazolines on β-amyloid aggregation kinetics. ACS Med. Chem. Lett..

[47] Reinke A.A., Gestwicki J.E. (2007). Structure–activity Relationships of Amyloid Beta-aggregation Inhibitors Based on Curcumin: Influence of Linker Length and Flexibility. Chem. Biol. Drug Des..

[48] Vistica D.T., Skehan P., Scudiero D., Monks A., Pittman A., Boyd M.R. (1991). Tetrazolium-based assays for cellular viability: A critical examination of selected parameters affecting formazan production. Cancer Res..

[49] Kedare S.B., Singh R.P. (2011). Genesis and development of DPPH method of antioxidant assay. J. Food Sci. Technol..

[50] Pohanka M., Hrabinova M., Kuca K., Simonato J.-P. (2011). Assessment of Acetylcholinesterase Activity Using Indoxylacetate and Comparison with the Standard Ellman’s Method. Int. J. Mol. Sci..

[51] Delfino R.T., Ribeiro T.S., Figueroa-Villar J.D. (2009). Organophosphorus Compounds as Chemical Warfare Agents: A Review. J. Brazil. Chem. Soc..

[52] Crivori P., Cruciani G., Carrupt P.A., Testa B. (2000). Predicting blood-brain barrier permeation from three-dimensional molecular structure. J. Med. Chem..

[53] Richter L., Munter L.-M., Ness J., Hildebrand P.W., Dasari M., Unterreitmeier S., Bulic B., Beyermann M., Gust R., Reif B. (2010). Amyloid beta 42 peptide (Aβ42)-lowering compounds directly bind to Aβ and interfere with amyloid precursor protein (APP) transmembrane dimerization. Proc. Natl. Acad. Sci. USA.

